# Tracking deep-sea internal wave propagation with a differential pressure gauge array

**DOI:** 10.1038/s41598-021-02721-1

**Published:** 2021-12-02

**Authors:** Chu-Fang Yang, Wu-Cheng Chi, Hans van Haren, Ching-Ren Lin, Ban-Yuan Kuo

**Affiliations:** 1 Earth System Science Program, Taiwan International Graduate Program (TIGP), Academia Sinica and National Central University, Taipei, Taiwan, ROC; 2 Institute of Earth Sciences, Academia Sinica, Taipei, Taiwan, ROC; 3 College of Earth Sciences, National Central University, Taoyuan, Taiwan, ROC; 4 Royal Netherlands Institute for Sea Research (NIOZ), P.O. Box 59, 1790 AB Den Burg, The Netherlands

**Keywords:** Ocean sciences, Solid Earth sciences

## Abstract

Temperature is used to trace ocean density variations, and reveals internal waves and turbulent motions in the deep ocean, called ‘internal motions.’ Ambient temperature detected by geophysical differential pressure gauges (DPGs) may provide year-long, complementary observations. Here, we use data from four DPGs fixed on the ocean bottom and a high-resolution temperature sensor (T-sensor) 13 m above the seafloor as a square-kilometer array deployed offshore ~ 50 km east of Taiwan facing the open Pacific Ocean to examine the impact of temperature on DPG signals related to internal motions. The DPG signals correlate with T-sensor temperature variations between 0.002 and 0.1 mHz, but have time shifts partially caused by slow thermal conduction from the ambient seafloor to the DPG chamber and partially by internal motion propagation time across the array. Applying beamforming-frequency-wavenumber analysis and linear regression to the arrayed T-sensor and DPG data, we estimate the propagating slowness of the internal motions to be between 0.5 and 7.4 s m^−1^ from the northwest and northeast quadrants of the array. The thermal relaxation time of the DPGs is within 10^3^–10^4^ s. This work shows that a systematic scan of DPG data at frequencies < 0.1 mHz may help shed light on patterns of internal wave propagation in the deep ocean, especially in multi-scale arrays.

## Introduction

Temperature is a crucial parameter to trace density variations in the ocean. Dynamical ocean internal motions and vertical turbulent water mixing are closely related to density distributions. However, density distributions are difficult to measure directly. As ocean density variations are a function of temperature, salinity, and pressure, these measurable parameters could be used to track density^1^. Temperature and pressure are relatively easily observed, but until now density has been unobservable and salinity can only be indirectly obtained by combining measurements of temperature and conductivity and to a lesser extent pressure. Combining measurements from different instruments with different characteristics sometimes deteriorates the precision of the quantity to be observed. To convert temperature to density, shipborne conductivity-temperature-depth profile measurements are used to build the local temperature-density relationship. Thus, temperature observations can be used as a tracer for density variations in the ocean at known pressures if the relationship is tight, e.g., van Haren and Gostiaux^2^. Compared with near-surface ocean waters, deep-ocean waters demonstrate relatively small temperature variations < 0.5 °C. However, slight changes of the temperature in the deep ocean may indicate internal waves and turbulent motions that may help to transport energy, suspended matter and nutrition for deep-ocean life, e.g., Thorpe^3^.

Internal waves, which propagate three-dimensionally in the ocean’s stable vertical density stratifications, induce temperature fluctuations in the deep-sea environment. These waves are ubiquitous in the ocean. Depending on their frequency and the rate of stratification, they can freely propagate, reflect, and scatter off seafloor topography^4^. The periods of internal waves range from several minutes, depending on the stratification, to a few days, depending on the latitude^5^. From short to long periods, internal waves can be induced by buoyancy oscillations, tides, and inertia-related geostrophic adjustments of passing atmospheric disturbances and Earth’s rotation. Among them, horizontal wavelengths of the longest-period inertial internal waves are observed O (10 km)^6^, related to the strength of the density stratification^7^.

When moored on the seafloor, temperature sensors (T-sensors) help to observe long-term internal motions in deep-sea environments. The time-series profiles from a moored vertical T-sensor string (T-string) are able to image vertical structures of the internal motions and to estimate the vertically spatial scale and energy of turbulent mixing induced by breaking of internal waves, e.g., van Haren^8^.

In geophysical research, seafloor pressure measurements from absolute and differential gauges have been applied to study seafloor geodesy^9,10^, tsunami propagation^11,12^, earthquake seismology^13,14^, and ocean surface infragravity waves^15^. The Cox-Webb differential pressure gauge (DPG)^16^ is a high-sensitivity pressure sensor usually integrated with an ocean bottom seismograph (OBS) for studying seafloor seismology. For example, seafloor compliance can be derived using data from co-installed DPGs and OBSs on the same frame to quantify how much seafloor ground displacement is induced by ocean surface waves, allowing us to study the rigidity of subsurface sediments^17–20^.

Despite wide applications of DPGs in seismology, amplitudes of the recorded signals are sensitive to the instrument response function that converts raw digital counts to physical units of pressure. Doran et al.^21^ found that the local environment of the deployment sites leads to variations in the response function, and in-situ calibration of DPGs helps to obtain accurate response information, which performs better than a general response function. However, this may limit the utility of the DPG data due to inaccurate quantification of pressure. Why an in-situ calibration is needed is an active research topic. In addition, pressure measurements of silicon oil-filled DPG sensors at frequencies below 3 mHz are sensitive to temperature changes^16^. The thermal effects from pressure-driven adiabatic processes and external temperature-induced conductive heat flow through the chamber walls can influence long-period DPG signals > 300 s^16^. There are several useful designs to avoid temperature effects in high-frequency pressure measurements^16^. An oil-filled acrylic plastic jacket surrounding the reference chamber contributes to thermal insulation for pressure signals at frequencies > 3 mHz, and a slow leak releases excessive pressure from the reference chamber about every 60 s to avoid large non-ambient-pressure-induced fluctuations in the chamber. Thus signals at > 3 mHz, typical for most earthquake seismological studies, e.g., An et al.^22^, are less affected by temperature. However, the impact of seafloor ambient temperature on field DPG signals at < 3 mHz has not been systematically documented so far.

**Figure 1:**
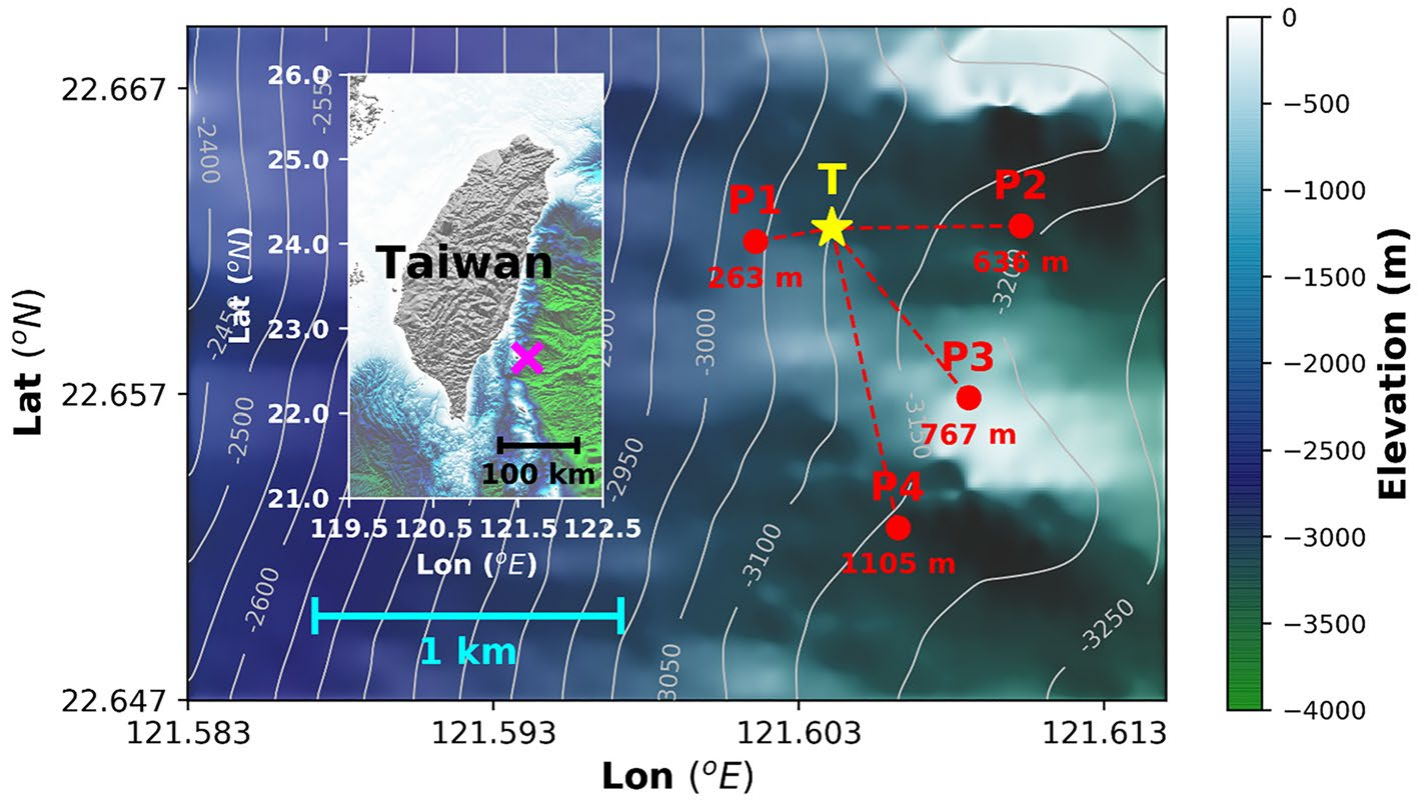
Location of the experimental site. A multi-instrument array within a 1 × 1 km footprint (magenta cross in insert) was deployed on continental slopes offshore eastern Taiwan. The array has a 200-m long vertical thermistor string (T-string, yellow star) with a Nortek AquaDopp acoustic current meter at 2936 m, and four Cox-Webb differential pressure gauges (DPG P1, P2, P3 and P4, red dots) attached to four broadband ocean bottom seismometers. Dashed red lines and numbers below the red dot show the shortest horizontal distances (with error ± 10 m) from the T-string to each DPG. Bathymetric maps with depths in [m] were made using the Matplotlib graphic tool (version 2.1.2; URL: matplotlib.org) for Python using 100 × 100 m resolution interpolated bathymetry data from a multibeam survey.

In this paper, we use data from a DPG array with a 1 × 1 km footprint, in addition to high-resolution T-sensor data from a mooring, to study DPG measurements of temperature fluctuations induced by deep-sea internal waves. The multi-instrument array was deployed on slopes at depths between 3000 and 3200 m offshore ~ 50 km east of Taiwan (Fig. 1) where internal waves are expected from the open Pacific Ocean. The array has four OBSs with horizontal station spacing of about 0.5–1 km and a single 200-m vertical T-string mooring with 101 T-sensors and a Nortek AquaDopp acoustic current meter with a pressure sensor. The concurrent observational period of the datasets was between September 1, 2017 and April 20, 2018. Each OBS has a Cox-Webb DPG. The DPGs (P1, P2, P3, and P4) are named sequentially from nearest to farthest from the T-string. The local inertial period of the deployment site at φ = 22.6° is about f = 2Ω sin φ ≈ 5.6 × 10^–5^ s^−1^ ≈ 0.77 cpd (cycle per day), approximately 0.0089 mHz. We analyzed the DPG variations at < 3 mHz with the data from a T-sensor at 3131 m to see how ambient temperature affects DPG waveforms, and to document temperature-induced variations from DPG records. We used DPG-derived ‘temperature signals’ in this spatially dense array to study events with significant internal motions, and to calculate the propagation speed and direction of the internal waves using beamforming-frequency-wavenumber (beamforming-FK) analysis^23,24^, henceforth ‘FK-analysis’ in short, and a linear regression method. In addition, the thermal relaxation time, the time for thermal conduction from a temperature pulse in the ambient environment to propagate to the DPG reference chamber, is estimated by temperature time delays between the T-sensor and four DPGs.

## Results

### Temperature-induced DPG variations

**Figure 2:**
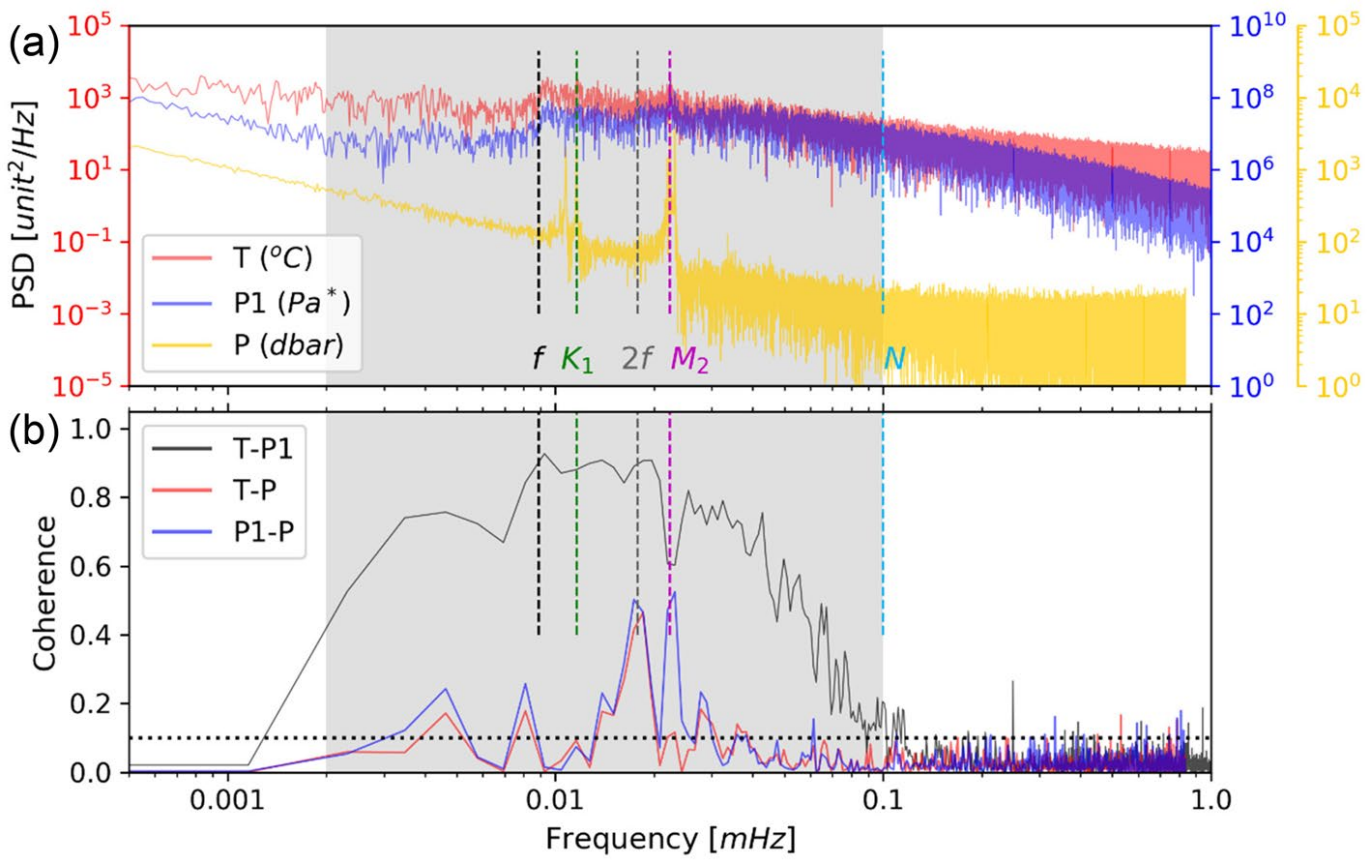
Spectral information. (**a**) Spectra of data from the T-sensor at 3131 m (T, red), from the differential pressure gauge (DPG) P1 at 3039 m (P1, blue, scale to the right), and from the pressure sensor at the current meter at 2936 m (P, yellow, scale to the far-right) for the period between September 1, 2017 and April 20, 2018. (**b**) Coherence between T and P1 (black), T and P (red), and P1 and P (blue). Dashed light blue, magenta, gray, green, and black lines mark the local buoyancy frequency (N), lunar semidiurnal frequency (M_2_), local double inertial frequency (2f), lunar diurnal frequency (K_1_), and local inertial frequency (f), respectively. Gray shades show the filtered bandwidth we used in this study.

The DPG variations between 0.002 and 0.1 mHz correlate well with T-sensor data, but do not correlate with the pressure data from the current meter. During the entire concurrent deployment period of all instruments, the pressure sensor on the current meter recorded strong diurnal and semidiurnal motions induced by ocean surface waves (Fig. 2a). The T-sensor and DPG P1 observed significant inertial and semidiurnal motions, and these two datasets show coherence > 0.1 (95% significance level) between 0.002 and 0.1 mHz (Fig. 2b). This frequency band includes periods of internal motions at the local inertial (f), the diurnal (K_1_), the double inertial (2f), and the semidiurnal (M_2_) frequencies, as well as the local buoyancy frequency N ≈ 0.1 mHz^25^. Although the bandwidth for coherence > 0.1 decreases as the DPG is further away from the T-sensor (Fig. S1), the coherence relations between the T-sensor and different DPGs are still similar at frequencies between 0.002 and 0.1 mHz (Fig. 2 and Fig. S1). The relations give a robust result that the long-period DPG variations are related to temperature variations induced by the internal motions.

**Figure 3:**
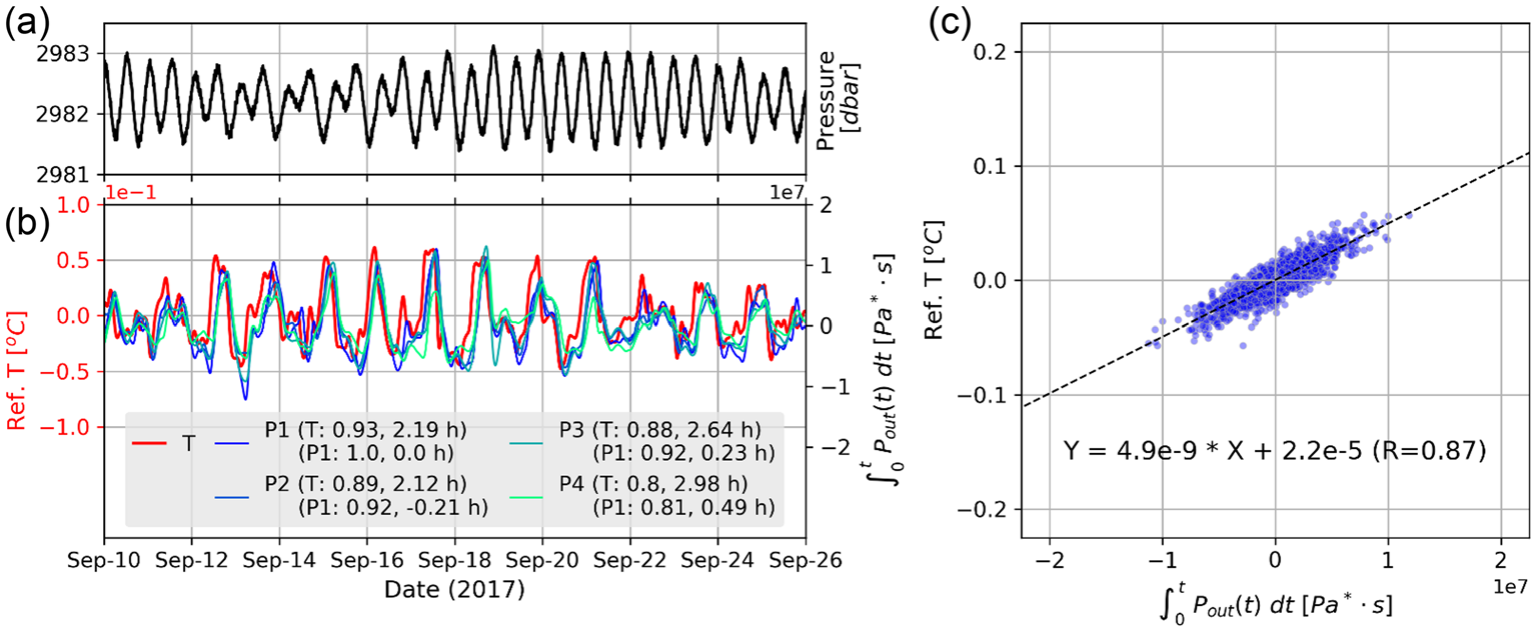
Time-domain comparison between filtered T-sensor and DPG data between September 10 and 25, 2017, with strong inertial internal motions. (**a**) Time series of pressure from the current meter. (**b**) Time series of referenced T-sensor and integrated DPG (DPG-T) data. For each DPG, we show the correlation coefficient and time shifts (in + hours) with respect to the T-sensor (T) and DPG-T P1 (P1), respectively. (**c**) Time shift-corrected DPG-T P1 variations against referenced T-sensor variations for the 15-day period in (**b**). The dashed black line shows the regression line and R is the Pearson correlation coefficient between the referenced T and DPG-T P1 datasets.

To examine the temperature-related data recorded by DPGs in the time domain, we use the significant coherence frequency band (0.002–0.1 mHz, henceforth ‘long period’ for this bandwidth) to filter the time series of the T-sensor and the DPG data for the following analysis. The DPGs are sensitive to the time derivative of temperature field for periods > 300 s^16^; thus, we integrate the long-period DPG variations for comparison with the temperature data. During a period of strong inertial internal motions with temperature variations of about 0.1 °C, the integrated long-period DPG variations correlate well with temperature variations (Fig. 3b), but show a time shift of a few hours with the T-sensor variations. Henceforth ‘DPG-T’ is informally defined for the temperature-related integrated DPG data. The pressure data from the current meter show clear diurnal and semidiurnal variations (Fig. 3a) that are not found in the DPG-T time series. The pressure variations O (10^4^ Pa) related to ocean surface tides are generally much greater than the pressure induced by internal tidal waves O (10^2^ Pa) in the deep ocean. However, surface tidal motions were barely visible in the DPG-T data. The different time/phase shifts among the DPGs also demonstrate that the DPG variations were not generated by ocean surface waves. The free propagating surface tidal waves, which have larger horizontal scales (O (10^3^ km)) and higher phase speed (O (10^2^ m s^−1^)), could arrive the DPG stations almost in-phase. Thus, we propose that the long-period DPG-T variations were related to the local environmental temperature, instead of ambient pressure variations. The time series at P1, which is the closest station to the T-sensor, has the highest correlation coefficient with the temperature variations, but the smallest time shift is found at P2. The correlation coefficients between the T-sensor and the DPGs decrease with increasing distance between them. Using the T-sensor data as a reference, we removed the time shifts from the cross correlations, and found that the DPG-T variations (Fig. 3c) at P1 show a linear relationship (R = 0.87) with the T-sensor variations.

The total time shifts between T-sensor and DPG-T data may be caused primarily by thermal relaxation time, the slow response of the DPG system to the ambient temperature, and partially by internal-motion propagation. The thermal relaxation time takes hours while the internal wave propagation across the array takes from 12 to 30 min. Such time shift relationships can be described as a simple equation of time shifts (see “Methods” section), and therefore the two different causes of the time shifts can be quantified using array analysis. We assumed that each DPG instrument behaves similarly, and has the same thermal relaxation time (${T}_{r}$). Thus, the time shifts induced by the propagation of internal waves can be quantified using the total time shift (${T}_{t}$) differences between the DPG-T variations that cancel out the constant ${T}_{r}$ in the DPG-T variations.

### Propagation of internal waves calculated from FK analysis

**Figure 4:**
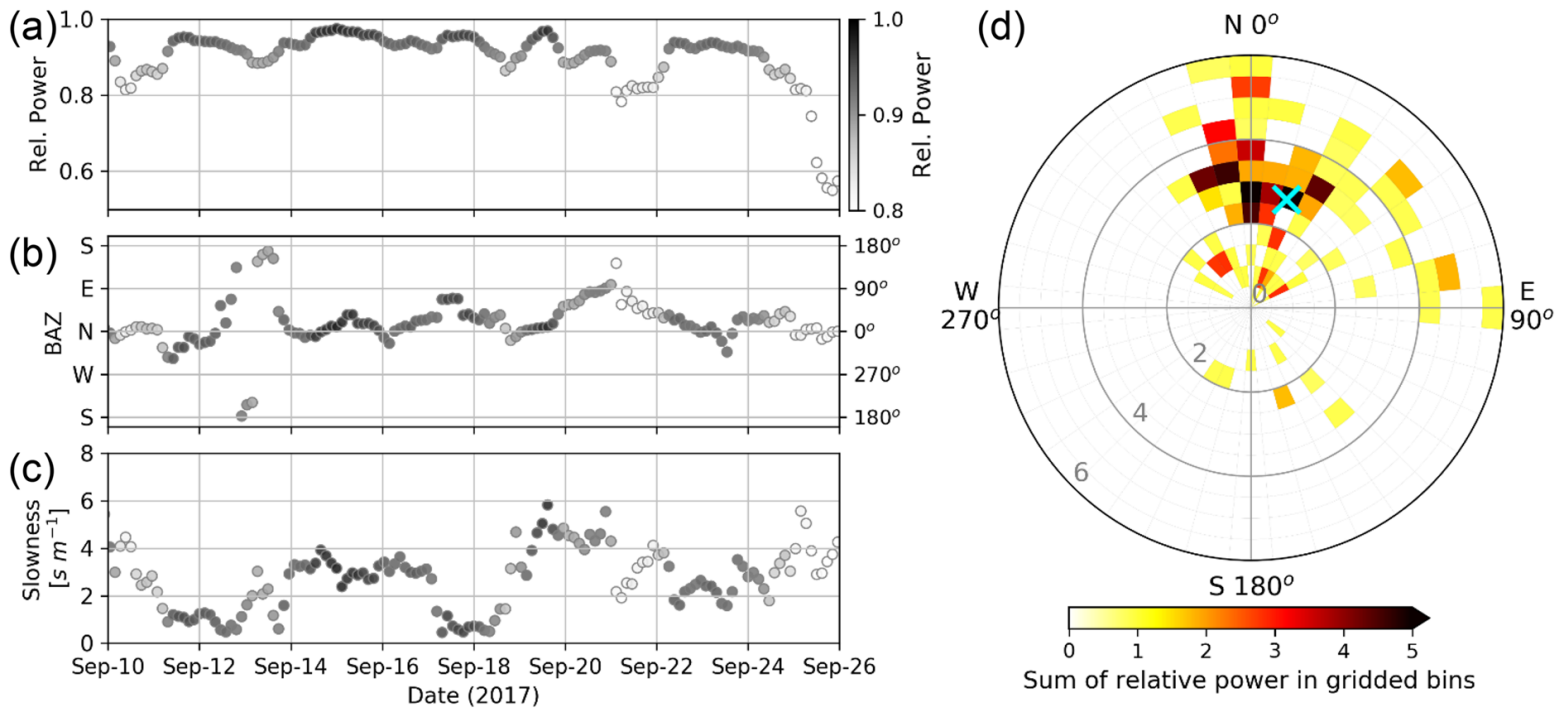
Directional phase propagation from data in Fig. 3b calculated using frequency-wavenumber (FK) analysis. Time series of (**a**) relative power, similar to goodness of waveform fits, (**b**) back azimuth (BAZ), and (**c**) slowness of propagation. (**d**) Polar plot of statistical BAZ and slowness (gray values in s m^−1^) with sum of relative power in gridded bins. Cyan cross shows the BAZ (data points in **b**) and slowness (data points in **c**) weighted average by the relative power (data points in **a**).

We used the DPG-T variations to estimate the direction and speed of propagating internal waves using FK analysis (Fig. 4, see “Methods” section). We analyzed an event with strong inertial internal motions recorded by the T-string between September 10 and 25 when Typhoon Talim was passing northwest of the array^25^. The time series of back azimuths (BAZs) and slowness from the FK analysis show that the internal waves were found to mainly come from the north to northeast of the array (Fig. 4b) and the propagating slowness varied by one order of magnitude between 0.5 and 6 s m^−1^ (Fig. 4c). A few waves propagated from the south of the array with slowness of 0.5–3 s m^−1^ between September 12 and 14. The varying slowness and BAZs of the internal waves might be caused by oscillating and sloshing of small-scale turbulent motions and high-frequency internal waves between topographic features in the array as found using other geophysical datasets^26^. The slowness of the strong waves from the north ranges between 2 and 4 s m^−1^, i.e. a propagation speed of about 0.25–0.5 m s^−1^ (Fig. 4d). This speed is consistent with the general phase speed of baroclinic inertial waves^27^. The horizontal wavelengths of the inertial internal waves were about 28–56 km.

We used FK analysis for other selected internal motion events (Table S1, see “Methods” section). There were 1–3 events per month with clear internal motions passing through the array. The internal waves were mostly from the northwest and the northeast quadrants of the array with weighted average propagating slowness ranging from 1.9 to 7.4 s m^−1^ (Fig. S2). The average thermal relaxation times are between 4000 and 8300 s in the selected 15 events (Table S1).

### Propagation of internal waves calculated from linear regression

**Figure 5:**
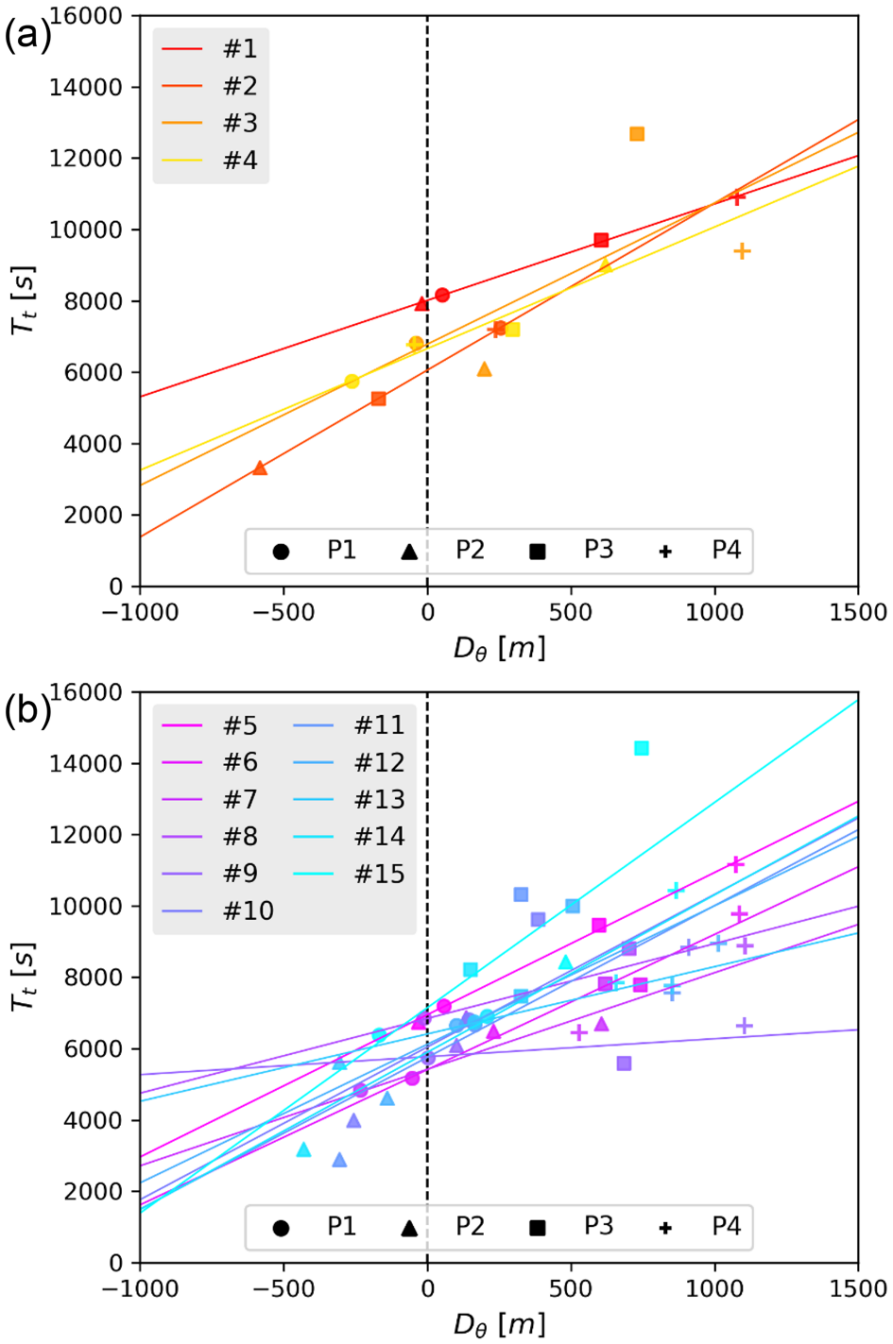
DPG-to-T-sensor horizontal distances along internal-wave propagation BAZ tracks against the time shifts between T-sensor and the DPG-T time series for selected events (Table S1). Events are defined by all pairs with correlation coefficient > 0.7. (**a**) Events with a coefficient > 0.8 and (**b**) with a coefficient between 0.7 and 0.79.

**Figure 6:**
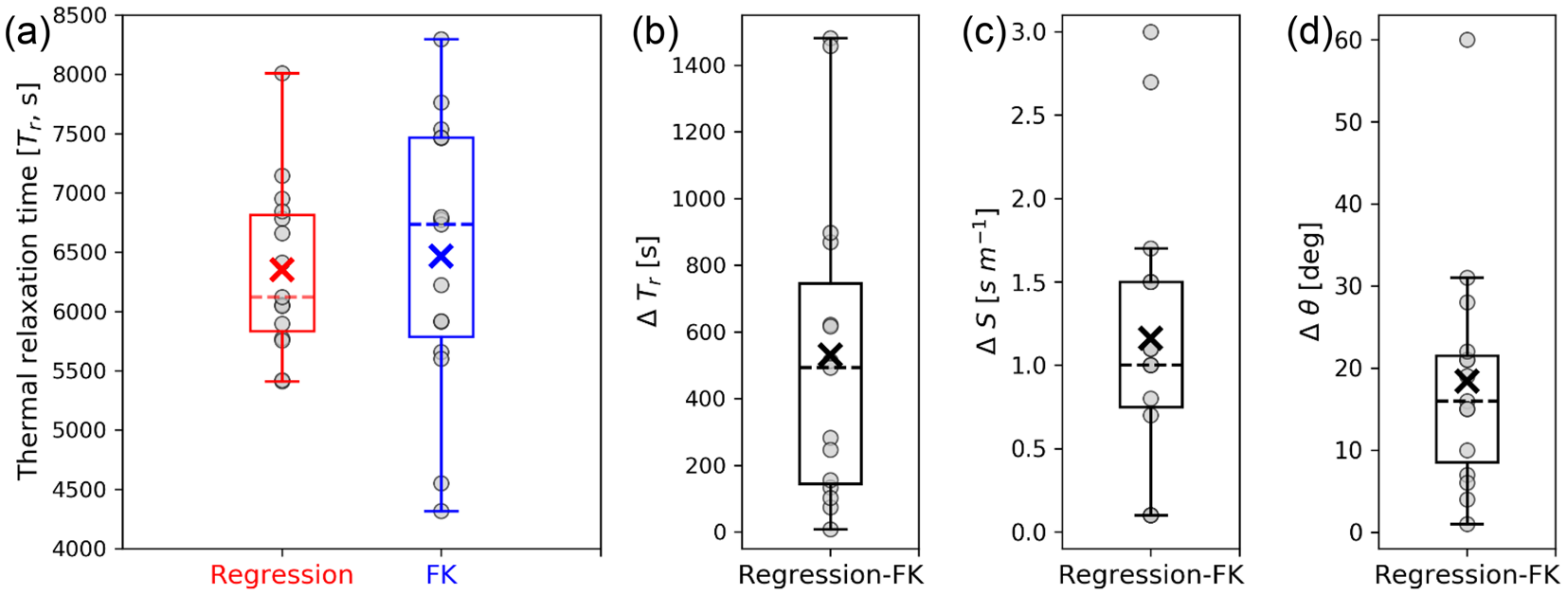
Comparing results from the two different methods for the events in Table S1. (**a**) Statistics of thermal relaxation time (T_r_) calculated by regression (red) and frequency-wavenumber ‘FK’ (blue) analyses. (**b**) Thermal relaxation time, (**c**) slowness (S), and (**d**) BAZ (θ) difference between estimation from regression and FK analysis in every event. Gray dots show data from the 15 events. The lower and upper lines on the sides of each box show the 25th percentile (Q1) and the 75th percentile (Q3) of the data, respectively. Median and mean values are shown with a dashed line and cross, respectively. The range between the bottom and top lines is within one and a half interquartile of Q1 and Q3.

We conducted root–mean–squared linear regression of the distributions of ${T}_{t}$ from cross correlation referenced by the T-sensor variations against the corresponding horizontal distance from the T-sensor along internal-wave propagation BAZ tracks to each station (${D}_{\theta }$). This is used to calculate the overall slowness and BAZ of the propagating internal waves for each event, as well as the thermal relaxation time (see “Methods” section). The regression functions show that the internal waves in the 15 events were mostly from the northwest and the northeast quadrants of the array with slowness ranging from 0.5 to 5.8 s m^−1^ (Table S1). The estimated thermal relaxation times in different events ranged from 5000 to 8000 s (Fig. 5), and when fitting the 15 events as a whole is about 6300 s (Fig. S3). The regression-estimated BAZs are mostly consistent with the FK-analysis-calculated BAZs, with discrepancy < 30° in most of the events (Fig. 6d). Although FK-analysis-estimated thermal relaxation times have a wider scatter ranging from 4250 to 8250 s (Fig. 6a), the regression-calculated slowness (O (1 s m^−1^)) and thermal relaxation times (O (10^3^ s)) are of the same magnitude as those calculated using the FK analysis (Fig. 6b,c).

## Discussion and conclusions

The high correlation between internal wave band data from DPG-T and T-sensor moored in the deep sea shows that ambient ocean temperature can affect long-period (0.002–0.1 mHz) pressure measurements of DPGs. This thermal effect can be caused not only by pressure-driven adiabatic processes but also by thermal conduction from the ambient environment exterior to the reference chamber. Applying the array analysis to T-sensor and DPG-T data, we can estimate the DPG thermal relaxation time caused by such thermal conduction. However, small-scale environmental difference and individual DPG configuration may cause uncertainties in quantifying accurate thermal relaxation time and a response transfer function between DPG-T and temperature data.

The pressure generated by thermal expansion of both reference chamber and fluid affects long-period DPG signals. In a DPG, the thermal expansion coefficient of the reference fluid, silicone oil, in the chamber is one order of magnitude larger than the coefficient of the reference chamber. According to the relation between the reference pressure of the DPG and ambient temperature^16^, there is about 1 Pa error if the temperature of the reference fluid and chamber changes by 0.8 μ°C assuming that the ambient pressure remains constant. Thus, the 0.1 °C difference from the typhoon-induced inertial motions in the deep-sea (Fig. 3b) might generate about 10^5^ Pa response, which is larger than the surface-wave-induced pressure changes of about 10^4^ Pa. Thus, the DPG data measured in this internal-motion-active area (Fig. 3b) barely show pressure variations from surface tidal motions. Such surface-wave pressure variations in the DPG-T variations are relatively small and thus are typically ignored. Comparatively, pressure variations driven by ocean-surface tides may dominate signals in the DPGs located at a flat deep seafloor in the open ocean where breaking internal waves are relatively inactive in weakly stratified waters. However, for the purpose of utilizing DPG-T responses as temperature data, it remains difficult to accurately quantify how much the amplitudes of the long-period variations are affected by pressure and temperature. In addition, the ingenious periodic slow leaking design of DPGs to avoid large excessive non-ambient-pressure-induced fluctuations might inadvertently generate additional long-period DPG response variations not directly related to the ambient environment. How the simultaneous increase and decrease of pressure affects the reference pressure of the DPG needs further examination. However, the DPG-T derived phase shift information is still useful for internal wave studies.

Sloshing waves and turbulence from waves breaking in the array may violate the plane wave assumption and cause changes in propagating speeds and directions of internal waves during the events. Compared to the overall ≥ 3-day duration of the internal motions fitted by the linear regression, the shorter duration of the 1.5-day time window with 10^4^-s time shifts used for the FK analysis may characterize shorter-period sloshing waves and turbulent motions induced by breaking of the waves in the array. Thus, the slowness and BAZs varied during the events. To reduce the issues on varying slowness and BAZs, it might be helpful to have arrays in an array with O (10–10^3^ m) horizontal separation, i.e. a multi-scale array, to cover a broader range of frequencies of internal waves and their associated turbulent motions. Nevertheless, the weighted average slowness and BAZs from the FK-analysis, which represent the main propagating internal waves, are of the same order of magnitude and consistent in direction with those calculated from the regression method.

For the regression analysis, because the horizontal scale of the internal motions is much larger than the vertical scale, we assumed that the internal waves are 2D plane waves instead of 3D waves in reality. However, this assumption may underestimate the slowness of the internal waves, so that the thermal relaxation times may be overestimated using the equation of time shifts. However, the varying thermal relaxation time in the events range over the same orders of magnitude (10^3^–10^4^ s). The periods of the DPG-T variations we analyze here are longer than the instrumental thermal relaxation time, demonstrating that the DPG may detect temperature fluctuations from internal waves.

The varying thermal relaxation times may also be due to differences between individual instruments. By allowing instrument-specific thermal relaxation times, we found statistically significant improvements in fitting of the regression model, indicating the DPGs may have their own thermal relaxation time (Text S1 and Fig. S3). On the other hand, assumption of allowing event-specific thermal relaxation times is not supported by the statistical examination (Text S1). The various time constants of each DPG do not have a systematic chronological trend except in the first three events (Fig. S4). Such scattering might be due to uncertainty of regression techniques and small-scale internal waves. Future studies are needed to better address this issue. For instance, analyzing data from exactly co-located T-sensors and DPGs may allow us to directly estimate the thermal relaxation time. Doran et al.^21^ demonstrated that the sensitivity and time constant of DPGs varied by at least a factor of two. Those variations are related to the depths at which instruments were deployed, seawater density, unpredictable behavior in the reference fluid under deep-sea mooring conditions, and slight imperfections of instrument components. Thus, they proposed that in-situ calibration of individual DPGs can improve accuracy of geophysical and oceanographic observations. This field dataset demonstrates how long-period DPG data are affected differently at each site by fluid motions, supporting the conclusions of Doran et al.^21^. Nevertheless, the high coherence of DPG signals among different stations (Fig. 3b) also suggest that slight imperfections of instrument components might not be an issue.

We applied array analysis to characterize the properties of propagating internal waves, without quantifying the pressure or temperature amplitudes associated with the waves; thus, the inaccuracies due to instrument-induced uncertainties are not a critical issue. Long-period DPG records can provide complementary observations to study deep-sea internal waves using array analysis. The varying thermal relaxation times during different internal wave events show that, in the future, multi-scale array observations may help to understand patterns of internal wave propagation over seafloor. In addition, further in-situ temperature calibration of the DPGs is needed by mounting a high-resolution temperature sensor on each OBS. Such datasets may help to build temperature response functions of the DPGs for further utilizing the data for physical oceanographic, seafloor geodetic, and ocean bottom seismological studies.

## Methods

### Instrument settings and data calibration

The Cox–Webb DPGs were built by the Institute of Earth Sciences of Academia Sinica. The output data were recorded using a well-tested in-house digitizer with a 100 Hz sampling rate. Instrument-caused diminished response is corrected using the general response function of the DPG and the sensitivity of the digitizer. The corrected DPG data at low-frequencies (< 3 mHz) may include a non-ambient-pressure-induced response (Fig. S5), e.g., temperature variation with time. Thus, we use the apparent pressure Pa*, instead of Pa, to discriminate fluctuations from ambient pressure.

The T-string had 101 T-sensors at 2-m intervals between 2937 and 3137 m that sampled data at a rate of 0.5 Hz and a Nortek AquaDopp acoustic current meter with pressure sensor at 2936 m sampling every 10 min. The T-sensors, with precision < 0.0005 °C and noise level < 0.0001 °C, were built by the Royal Netherlands Institute for Sea Research (NIOZ)^28^. Here, we used data from the T-sensor at 3131 m, just 13 m above the seafloor, to minimize the influence of high-frequency temperature fluctuations caused by turbulent disturbances higher in the water column.

### Time shift equation

The total time shifts between T-sensor and DPG-T variations can be described simply by the following equation:

$${T}_{t}=S\times {D}_{\theta }+{T}_{r}$$

${T}_{t}$ is the total time shift between the T-sensor and DPG-T variations. $S\times {D}_{\theta }$ is the time shift caused by the propagation of internal waves. $S$ is the horizontal slowness of the internal wave. ${D}_{\theta }$ is the horizontal distance between DPG and T-sensor along internal-wave propagation BAZ tracks. ${T}_{r}$ is the thermal relaxation time.

### Internal wave events

Fifteen internal wave events (Table S1) were identified by cross correlation using a 3-day time window with 1-day shifting step for every pair of T-sensor and DPG-T variations with correlation coefficient > 0.7 to ensure that events with duration > 3 days can be integrated to the same event. The events have internal motion lasting ≥ 3 days, including ≥ 2 cycles of the local inertial periods and ≥ 3 cycles of tidal periods.

### Beamforming-frequency-wavenumber analysis

Beamforming-FK analysis transforms waves to frequency-wavenumber relations before ‘beams’ and stacks them over a predefined grid for slowness points. It then calculates the power of a beam with different slowness and BAZs of an assumed incoming plane wave crossing the array, allowing us to find the preferred model of apparent slowness and BAZs. The slowness and BAZs of the internal waves in each event were calculated from the long-period DPG-T variations using a 1.5-day time window, which includes the inertial and tidal periods, and a 10^4^-s window shift.

### Root-mean-squared linear regression method

Root-mean-squared linear regression of the distributions of ${T}_{t}$ from cross correlation referenced by the T-sensor variations and ${D}_{\theta }$ at each station can be used to calculate the overall slowness and BAZ of the propagating internal waves for each event. We searched the ${D}_{\theta }$ at each station using a grid search of DPG to internal-wave BAZ every 1° within 360° to find the one that gives the maximum Pearson correlation coefficient R. According to the equation of time shifts, the slope and intercept of the regression line are the average horizontal slowness of the propagating internal wave and the thermal relaxation time, respectively.

## Supplementary Information

Supplementary Information.

## Data Availability

Data associated with the present paper are available from the TEC Data Center (https://tecdc.earth.sinica.edu.tw/WAV/GreenIslandArray).
