# Supplementary material for: Tracking deep-sea internal wave propagation with a differential pressure gauge array
Source: Sci Rep. 2021 Dec 2;11:23311. doi: 10.1038/s41598-021-02721-1 (PMC8639723; doi:10.1038/s41598-021-02721-1)
Supplement: Supplementary file 1 — Supplementary Information. [file 41598_2021_2721_MOESM1_ESM.docx]

***Scientific Reports***

Supplementary Information for

**Tracking deep-sea internal wave propagation with a differential pressure gauge array**

Chu-Fang Yang^1,2,3^, Wu-Cheng Chi^2^, Hans van Haren^4^, Ching-Ren Lin^2^, Ban-Yuan Kuo^2^

^1^ Earth System Science Program, Taiwan International Graduate Program (TIGP), Academia Sinica and National Central University, Taipei, Taiwan

^2^ Institute of Earth Sciences, Academia Sinica, Taipei, Taiwan

^3^ College of Earth Sciences, National Central University, Taoyuan, Taiwan

^4^ Royal Netherlands Institute for Sea Research (NIOZ), P.O. Box 59, 1790 AB Den Burg, the Netherlands

**Contents of this file**

Text S1

Figures S1 to S5

Table S1

**Introduction**

The supplementary information contains details of the F-test (Text S1), five figures (Figure S1 to S5) and a table (Table S1) to complement our interpretation in the study.

**Text S1**

**Partial F-test for testing single and multiple variables of the thermal relaxation time**

We conducted the partial F-test to statistically examine if the DPG thermal relaxation time ($T_{r}$) depends on each specific DPG, and to see if the DPGs degrade over time, causing time-dependent/event-specific $T_{r}$. There are totally 60 data points, from internal-wave propagation distance ($D_{\theta}$) against total time shifts ($T_{t}$), used for obtaining single and multiple $T_{r}$ variables by fitting different groups of the data. These 60 data points are from 4 DPGs recording15 selected events. To obtain single $T_{r}$ variable for the reduced model, we fitted all 60 data points (Figure S3). For comparing the full model with instrument-specific $T_{r}$ to the reduced model, we fitted 15 event data for each DPG to obtain four $T_{r}$ variables (Figure S3). For comparing the other full model with event-specific $T_{r}$ to the reduced model, we fitted 4 DPG data for every event to obtain fifteen $T_{r}$ variables (shown in the main text and Figure 5). The partial F-value is defined as

$$F=\frac{\left[ {SS}_{1}-{SS}_{m} \right]/p}{{SS}_{m}/\left( n-m \right)}$$

where $SS_{1}$ and $SS_{m}$ are sum of squares of residuals for single- and multiple-variable models, respectively. $p$ is the number of variables removed from the reduced, single-variable model ($p=m-1$, $p=3$ for four-variable model and $p=14$ for fifteen-variable model), $n$ is the total observation in the dataset ($n=60$), and $m$ is the number of variables of the full, multiple-variable models ($m=4$for four-variable model and $m=15$ for fifteen-variable model). The residuals for $SS_{1}$, $SS_{4}$, and $SS_{15}$ are the difference between time discrepancies ($T_{t}-S\times D_{\theta}$) and their corresponding $T_{r}$ variables. $S\times D_{\theta}$ are from the same data estimated from grid search for the selected 15 event.

The partial F value by allowing instrument-specific $T_{r}$ is 3.52 and the critical F value for the degrees of freedom of $p=3$ and $n-m=56$ at 95% confidence level is 2.76. On the other hand, for event-specific $T_{r}$, the partial F value is 1.48 and the critical F value for the degrees of freedom of $p=14$ and $n-m=45$ at 95% confidence level is 1.84. The partial F values indicate that allowing individual variable for each DPG (four $T_{r}$ variables) makes significant improvements, but the fifteen event-specific $T_{r}$ variables do not significantly improve the fitting of regression model. Namely, the DPGs tend to have individual $T_{r}$, and the $T_{r}$ do not have significant trend over time (with events).


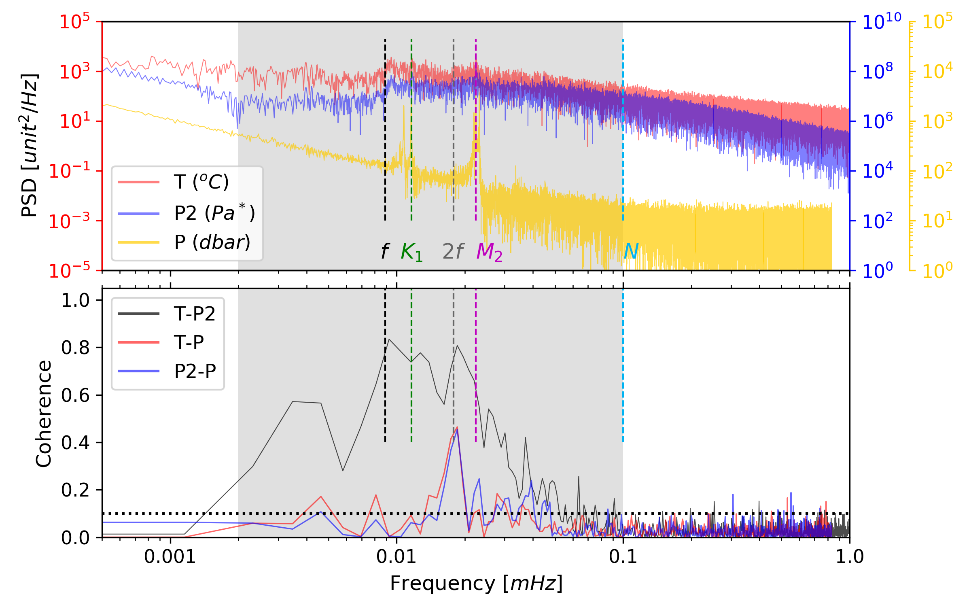

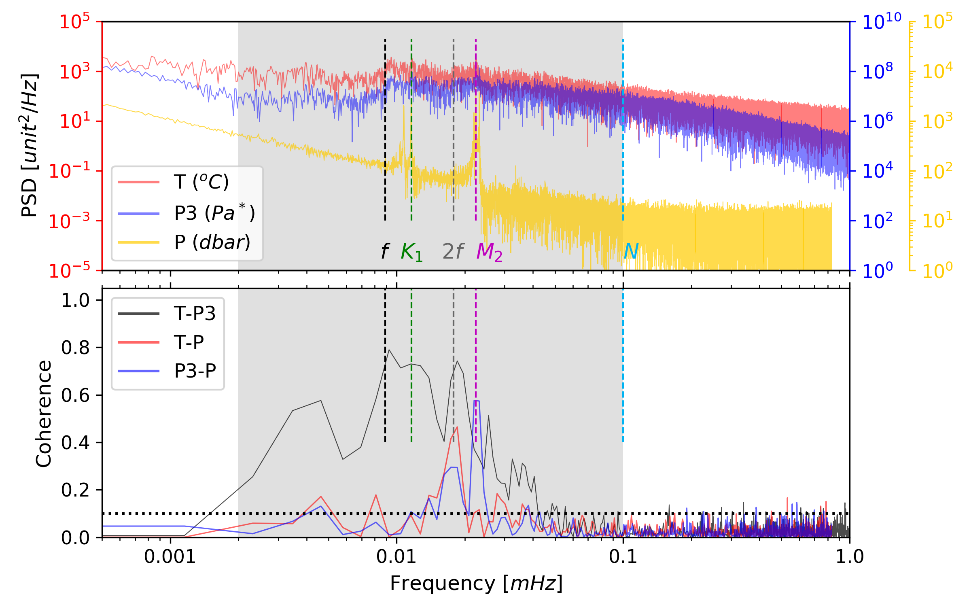

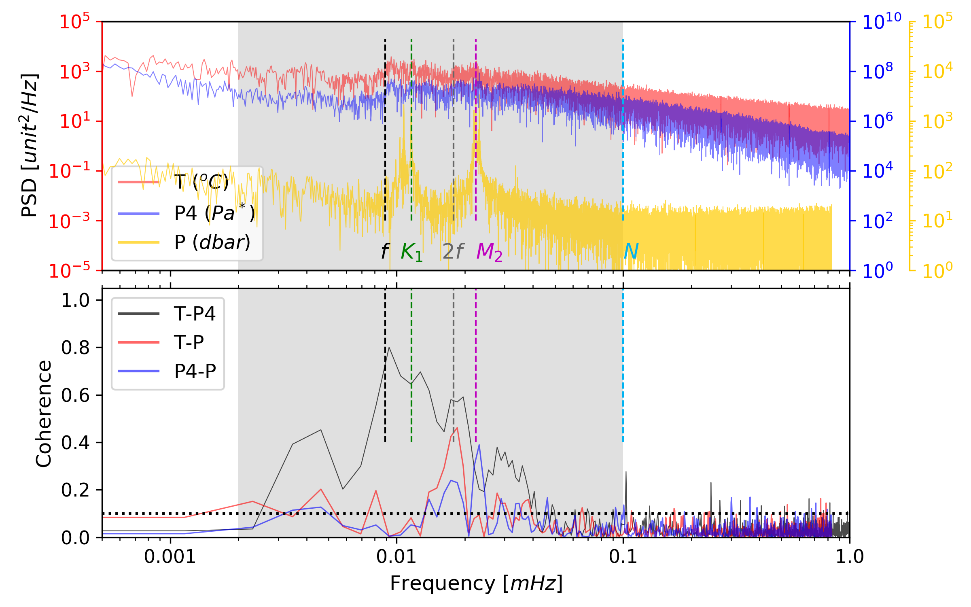


(a)

(c)

(b)

Figure S1. As Fig. 2, but from DPG (a) P2, (b) P3, and (c) P4. Spectra of the temperature, pressure, and DPG P4 data in (c) are for period between September 1, 2017 and April 2, 2018. Coherences between the T-sensor and different DPGs are similar between 0.002 and 0.1 mHz, but with fast fall off at higher frequencies (> 0.05 mHz). Bandwidth of the coherence becomes narrower as the DPGs are away from the T-sensor, showing that small-scale (< station distances) internal waves were active between the stations.


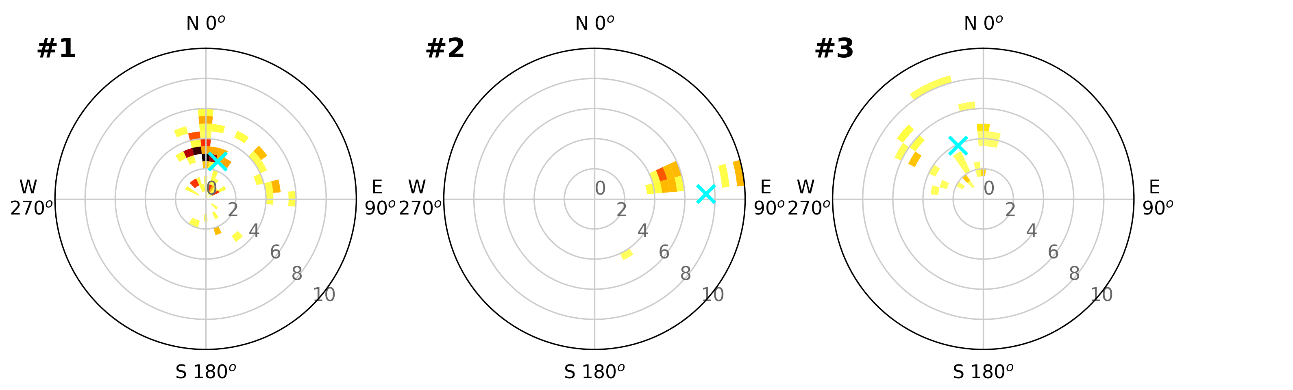

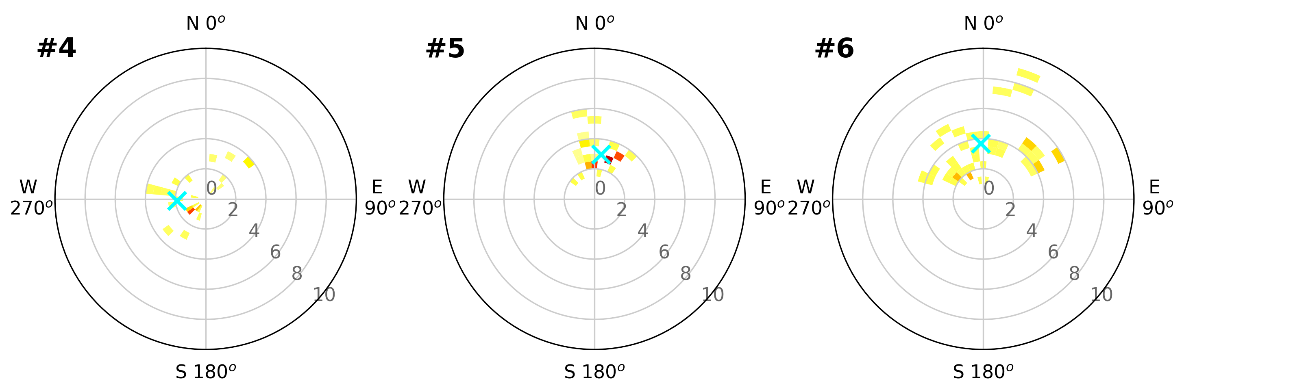

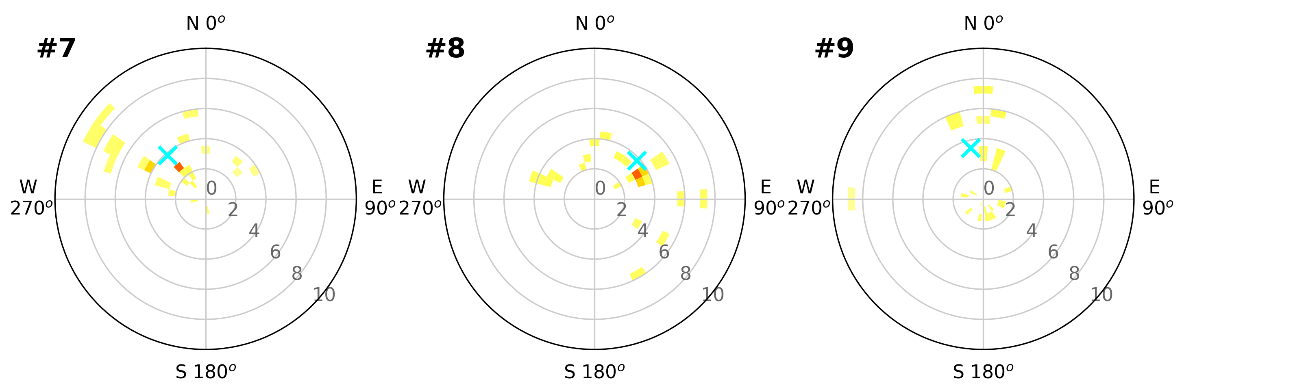

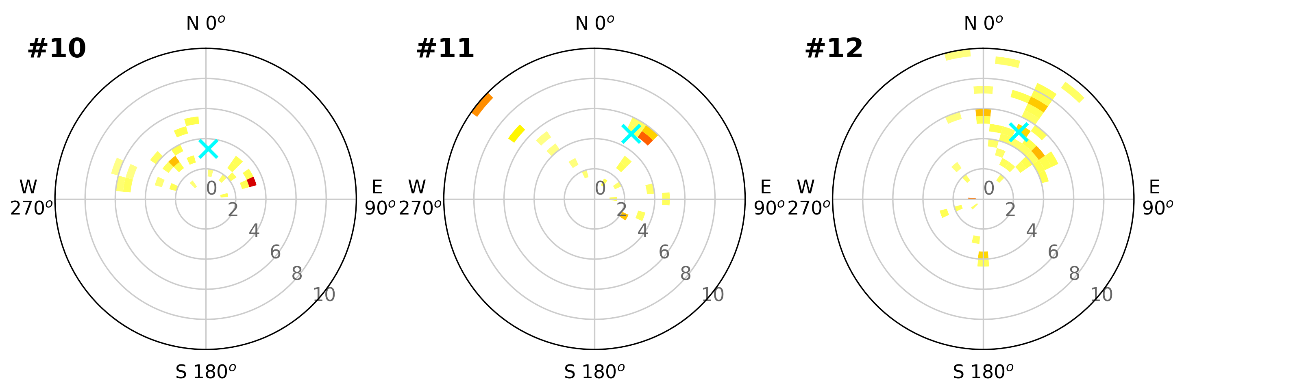

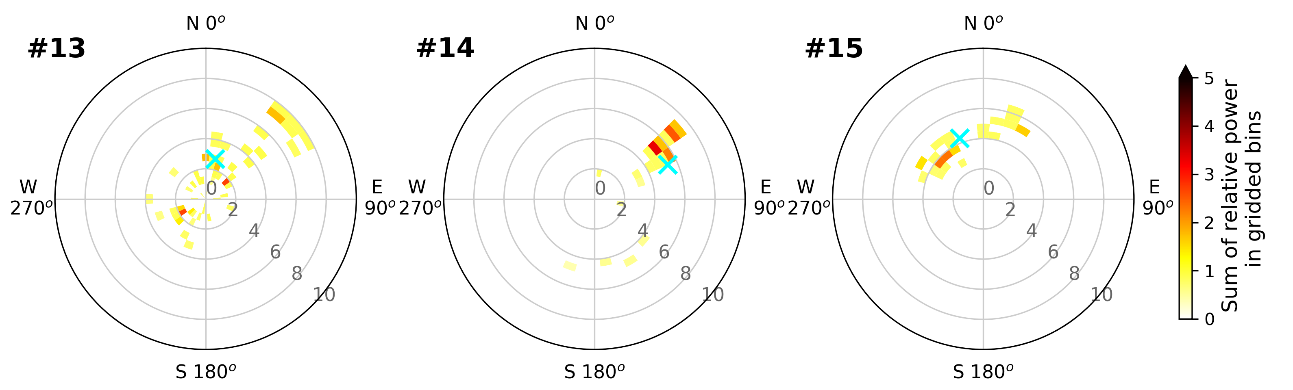


Figure S2. As Fig. 4d, but for the periods during the 15 events shown in Table S1.


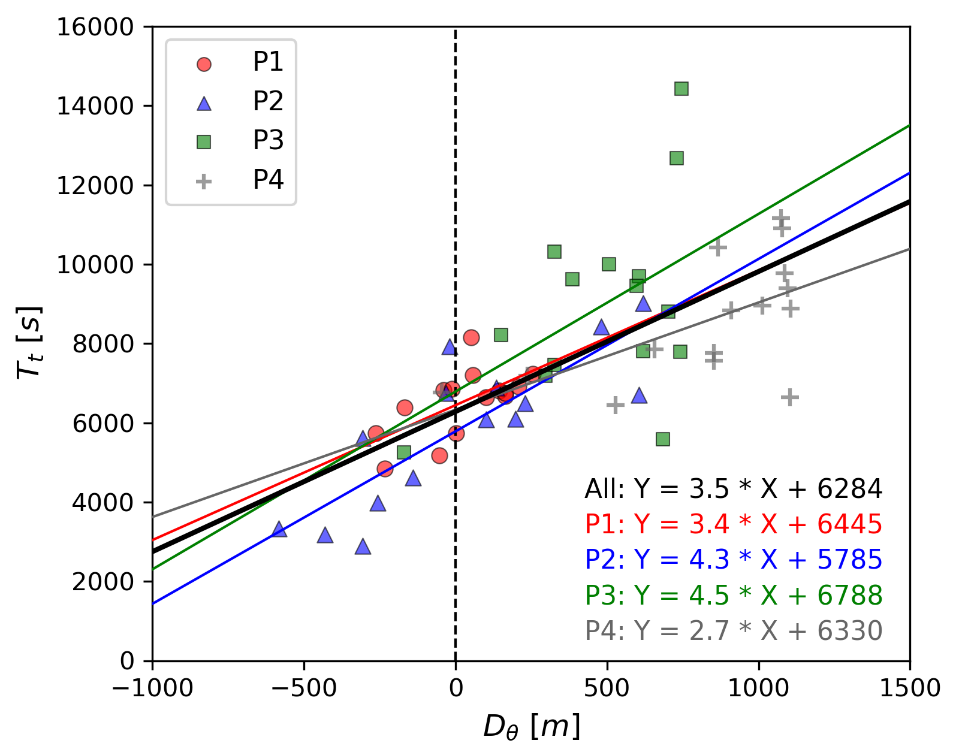


Figure S3. As Fig 5, but fitting all data in Fig 5a and 5b (shown with black line), and fitting event data for DPG P1, P2, P3, and P4 (shown with red, blue, green, and gray lines, respectively).


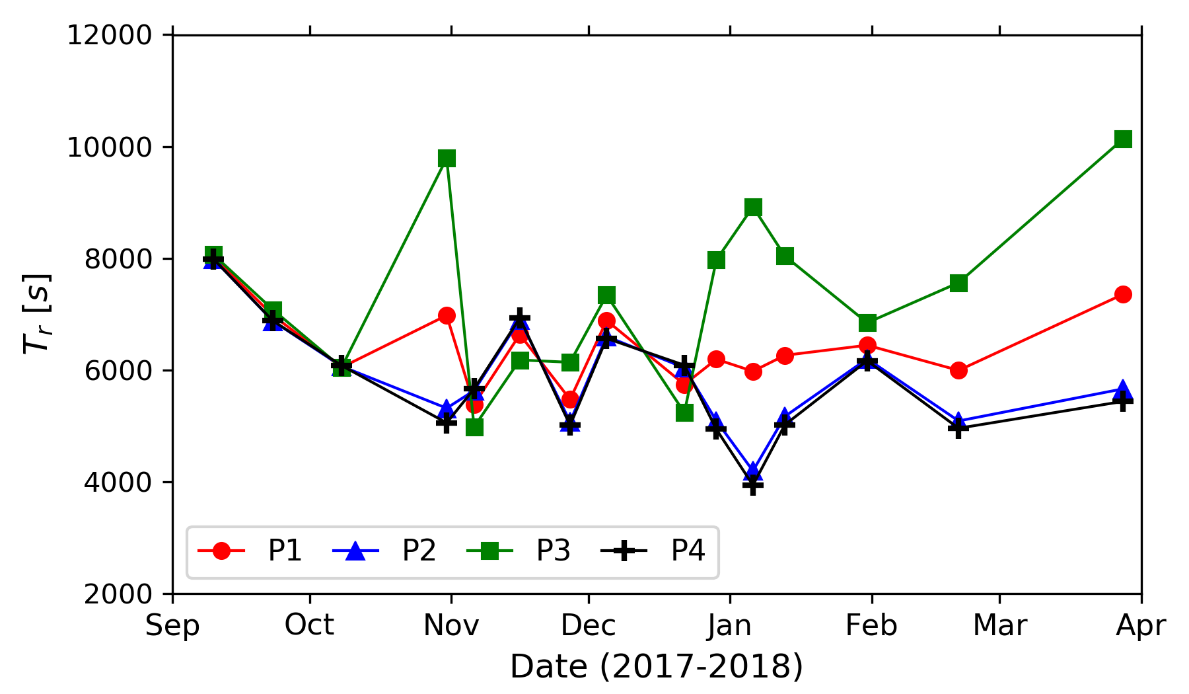


Figure S4. Time series of regression-calculated individual thermal relaxation time (T_r_) of four DPGs in 15 events. T_r_ of each DPG is calculated by the total time shifts (T_t_) and the regression-estimated slowness and BAZ of internal wave progression for each event in Table S1. Except the first three events, the T_r_ does not show a systemic trend with time. The scattering T_r_ may be caused by inner-array small-scale internal waves and turbulence, whose slowness and BAZ also vary for each event.


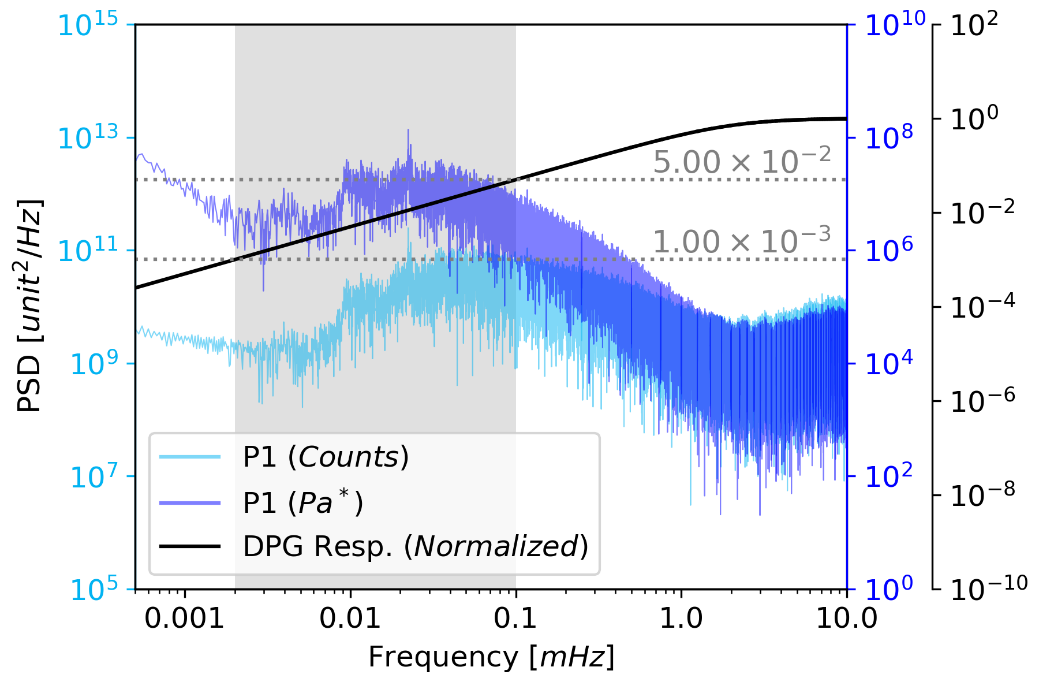


Figure S5. Amplification/correction of differential pressure gauges (DPGs). Spectra of raw (digital counts, light blue) and response-calibrated (Pa*, dark blue) data from DPG P1 at 3039 m between September 1, 2017 and April 20, 2018. The black line shows the normalized response function of the DPG, and the dashed gray lines show the normalized response (power spectral density, PSD) at corner frequencies 0.002 and 0.1 mHz of the filter bandwidth (gray shades). The unit Pa* is the Pascal calibrated by deconvolution of the DPG response function in the frequency domain which amplifies the diminished responses due to low sensitivity at frequencies < 2 mHz, approximately the low-corner frequency of the flat response.

Table S1. Details of the internal wave events. The events were selected under the criterion that the T-sensor-DPG correlation coefficient was > 0.7 at every DPG in a 3-day time window that is shifted in time by 1 day. Events #1–4 and events #5–15 have correlation coefficients > 0.8 and between 0.7 and 0.79, respectively.

| Event  (YYYY/MM/DD) | Regression analysis | | | FK analysis | | |
| --- | --- | --- | --- | --- | --- | --- |
|  | BAZ | Slowness  (S, s m^-1^) | Thermal relaxation time  (T_r_, s) | Weighted BAZ | Weighted slowness  (S, s m^-1^) | Avg. thermal relaxation time  (T_r_, s) |
| 1. 2017/09/10-2017/09/21 | 1° | 2.7 | 8012 | 17° | 2.6 | 8295 |
| 1. 2017/10/08-2017/10/11 | 66° | 4.7 | 6058 | 87° | 7.4 | 7539 |
| 1. 2017/10/31-2017/11/03 | 341° | 4.0 | 6783 | 334° | 3.9 | 6775 |
| 1. 2017/11/16-2017/11/19 | 256° | 3.4 | 6660 | 266° | 1.9 | 6734 |
| 1. 2017/09/23-2017/09/26 | 2° | 4.0 | 6951 | 8° | 3.0 | 7464 |
| 1. 2017/11/06-2017/11/11 | 338° | 3.8 | 5412 | 357° | 3.7 | 5659 |
| 1. 2017/11/27-2017/11/30 | 287° | 2.7 | 5422 | 318° | 3.8 | 4553 |
| 1. 2017/12/05-2017/12/08 | 347° | 2.1 | 6847 | 47° | 3.8 | 7469 |
| 1. 2017/12/22-2017/12/25 | 350° | 0.5 | 5774 | 346° | 3.5 | 4316 |
| 1. 2017/12/29-2018/01/01 | 23° | 4.3 | 6046 | 2° | 3.3 | 5913 |
| 1. 2018/01/06-2018/01/09 | 28° | 4.3 | 5757 | 29° | 5.0 | 5602 |
| 1. 2018/01/13-2018/01/19 | 12° | 3.9 | 6121 | 27° | 5.0 | 6223 |
| 1. 2018/01/31-2018/02/07 | 28° | 1.9 | 6412 | 13° | 2.7 | 5920 |
| 1. 2018/02/20-2018/02/23 | 42° | 4.4 | 5899 | 64° | 5.4 | 6797 |
| 1. 2018/03/28-2018/03/31 | 310° | 5.8 | 7146 | 338° | 4.3 | 7762 |
